# A wireless sensor network node fault diagnosis model based on belief rule base with power set

**DOI:** 10.1016/j.heliyon.2022.e10879

**Published:** 2022-10-07

**Authors:** Guo-Wen Sun, Wei He, Hai-Long Zhu, Zi-Jiang Yang, Quan-Qi Mu, Yu-He Wang

**Affiliations:** a Harbin Normal University, Harbin, 150025, China; b Rocket Force University of Engineering, Xi'an 710025, China; c Heilongjiang Agricultural Engineering Vocational College, Harbin, 157041, China

**Keywords:** Fault diagnosis, Wireless sensor network, Belief rule base, Power set

## Abstract

Wireless sensor network (WSN) is inevitably subject to node failures due to their harsh operating environments and extra-long working hours. In order to ensure reliable and correct data collection, WSN node fault diagnosis is necessary. Fault diagnosis of sensor nodes usually requires the extraction of data features from the original collected data. However, the data features of different types of faults sometimes have similarities, making it difficult to distinguish and represent the types of faults in the diagnosis results, these indistinguishable types of faults are called ambiguous information. Therefore, a belief rule base with power set (PBRB) fault diagnosis method is proposed. In this method, the power set identification framework is used to represent the fuzzy information, the evidential reasoning (ER) method is used as the reasoning process, and the projection covariance matrix adaptive evolution strategy (P-CMA-ES) is used as the parameter optimization algorithm. The results of the case study show that PBRB method has higher accuracy and better stability compared to other commonly used fault diagnosis methods. According to the research results, PBRB can not only represent the fault types that are difficult to distinguish, but also has the advantage of small sample training. This makes the model obtain high fault diagnosis accuracy and stability.

## 1 Introduction

A wireless sensor network (WSN) is a new type of information acquisition and processing network. It consists of a large number of low-power sensor nodes. The sensor nodes communicate through a wireless network. WSN has been widely used in mechanical parameter detection, industrial monitoring, mine safety, medical and health, environmental monitoring and other industries. With the rapid development of Internet of Things (IOT) industry, WSN is also widely used in smart home field. The application scenarios of WSNs usually require real-time and reliable data collection. However, the working environment of industrial WSN is harsh, usually in a high temperature and high pressure environment. In the field of IoT, it is difficult to avoid communication interference between multiple networks. Moreover, with the accumulation of the working time of WSN node, the possibility of its failure gradually increases. Therefore, to ensure the real-time reliability of the data collected by WSN and to grasp the fault of WSN node in time, the fault diagnosis of WSN node is particularly important [1].

Commonly used WSN fault diagnosis methods can be divided into three types: model analysis methods, data-driven methods and hybrid information-based methods. First, some of the methods based on model analysis are not accurate due to the complexity of the system and the relative simplicity of the model. Second, data-driven based approach needs a large number of uniform fault samples is required to obtain better diagnosis. Moreover, whether the approach is model-based, data-driven or mixed-information, there is a common problem. Fault types that are difficult to distinguish during diagnosis, i.e., ambiguous information, cannot be represented, this affecting the accuracy of fault diagnosis.

The proposed method of WSN node fault diagnosis based on power set belief rule base (PBRB) has two advantages. Firstly, the model can express the fuzzy information of fault classification caused by the similarity of data features in power set. Second, the setting of the initial parameters of the model is derived from expert knowledge, which can effectively improve the accuracy of model diagnosis and has little dependence on the number of training samples.

The basic structure of the paper is as follows. Section 2 is related work, which introduces the common methods and existing problems of WSN fault diagnosis. In Section 3, the problems of WSN node fault diagnosis are formulated, and then a new fault diagnosis model is constructed based on the PBRB. In Section 4, the implementation process of the model is designed. In Section 5, the case study is constructed to verify the effectiveness of the model. In Section 6, the results of the case study are discussed. In Section 7, the content of the paper is summarized, and future work is planned.

## 2 Related work

Due to the wide application of WSN and the importance of WSN node fault diagnosis. WSN node fault diagnosis is the research direction of many scholars.

The first type of method is based on model analysis. It is defined as simulating the cognitive process of things, which includes expert system-based models, fuzzy logic-based models, decision tree-based models and hypothesis testing-based models [2, 3, 4, 5, 6, 7]. Febriansyah, II. Saputro, WC. et al. studied and implemented a combination of multiscale principal component analysis (MSPCA) and decision trees to detect fault data from WSN and classify faults [4]. The different factors of sensor nodes are analyzed, and a fault diagnosis method for heterogeneous WSN based on fuzzy logic is proposed by M. Masdari et al. [5]. Laiou, A. et al. constructed an autonomous fault diagnosis model in a WSN based on a decision tree [6]. Sun, QY. Sun, YM. et al. proposed a method for multi-classification of WSN nodes based on a combination of recursive principal component analysis (RPCA) and support vector data description (SVDD) [7]. This type of method does not rely on fault samples and has a wide range of applications. However, the model is affected by the complex environment, the modeling accuracy of the model is low, and the model learning ability is poor.

The second type of method is the data-driven method. It is defined by learning the fault samples [8]. The types of methods mainly include neural network-based (NN) models, extreme learning machine-based models and extremely randomized trees [9, 10, 11, 12, 13, 14, 15]. An automatic fault diagnosis model based on back propagation neural network (BPNN) was proposed by Swain, R. R. Khilar, P. M. et al. to determine multiple fault types of WSN hard and soft faults [9]. Gui, W., Lu, Q., Su, M., & Pan, F. proposed a convolutional neural network based on the optimization of the Fireworks algorithm for fault diagnosis of WSN nodes [10]. By considering the improved belief function fusion method, an enhanced recurrent ELM-based method for WSN fault diagnosis was proposed by A. Javaid et al. [14]. The data-driven method is currently the main fault diagnosis technology of WSN, which has the advantage of high model accuracy. However, these methods rely on the integrity of historical data, there is no causal relationship in the modelling process, the initial parameters of the model are set randomly, and the random parameters are largely incompatible with WSN mechanism, which can lead to limited diagnostic accuracy of the model.

The third type of method is the hybrid information-based method. Defined by learning qualitative knowledge and quantitative data [16]. In this type of method, the Markov-based model, Bayesian network-based model and belief rule base (BRB) model are often used. Zhao, Qun. proposed a multi-channel information fusion method based on coupled hidden Markov models for fault diagnosis of mechanical equipment [17]. Emperuman, M. et al. proposed a continuous density hidden Markov model, combined with neural networks, for fault classification of sensor devices in WSNs [18]. A diagnostic model based on Markov transition fields and deep residual networks was proposed by Yan, J., Kan, J. et al. [19]. Through the mapping relationship between fault trees and Bayesian networks, Chunhua Zhang et al. constructed a fault diagnosis model for Bayesian networks [20]. A fault diagnosis method using Bayesian networks as a model bridge was proposed through the mapping relationship between fuzzy fault trees and BRBs by Cheng, X., Liu, S. et al. [21]. However, these methods have high modelling requirements. The validity and accuracy of the model can only be guaranteed when the type of fault being diagnosed is identified and the characteristics are well defined.

BRB is an excellent modeling method for complex systems based on hybrid information [22]. The BRB model is built using expert knowledge, and the BRB model parameters are trained using historical data. In addition, all kinds of uncertain information can be effectively processed by BRB, including randomness, fuzziness, uncertainty, and inconsistency [23]. The theory of BRB has gradually improved, and BRB are widely used in many fields, such as medical decision-making [24], fault diagnosis [25], and safety assessment [26].

In the study of WSN node fault diagnosis, WSN node fault types are diagnosed by the data features extracted from the original collected data. However, in engineering practice, the data features of different types of faults in a specific interval are similar, which makes it difficult to diagnose fault type. These faults, which cannot be clearly diagnosed, are called ignorance information including local ignorance and global ignorance. Therefore, WSN node fault diagnosis model should have the ability to describe the ignorance information [27]. However, the discriminative framework of the belief rule base cannot effectively represent this ignorance information. In the latest research, to solve that BRB cannot effectively describe local ignorance, ZJ. Zhou et al. extended BRB with the power set framework [28]. The ignorance information of complex systems can be more efficiently represented using a power set identification framework.

Through the above analysis, in this paper, the PBRB is proposed as a fault diagnosis method for WSN nodes. This method has several advantages. First, both qualitative and quantitative information can be used as inputs of the model to form IF-THEN rules, which are suitable for the modelling requirements of large and complex systems. Second, it is possible to represent information about fault types that are ambiguous due to similar data features, improving the accuracy of fault diagnosis. Finally, compared with other data-driven methods, this method is more consistent with the working mechanism of the diagnosed device. It sets parameters according to expert knowledge and enhances the interpretability of the model while improving the diagnostic accuracy.

## 3 Problem formulation

In this section, the problems in fault diagnosis for WSN nodes are formulated, and a new WSN node fault diagnosis model is constructed based on the PBRB.

### 3.1 Problem formulation of WSN node fault diagnosis

**Figure 1:**
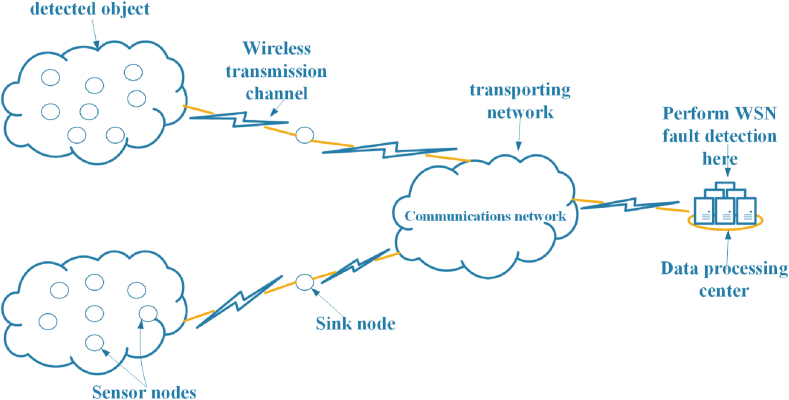
WSN fault diagnose in the data processing center.

In the WSN, a large number of sensor nodes are randomly distributed in or near the monitoring area, which can form a network in a self-organizing manner. The data detected by the sensor nodes are transmitted to the sink node through the self-organizing network. The sink node transmits the data to the management node, that is, the data processing center, through Internet or satellite communication. Therefore, it is an effective solution to implement WSN node fault diagnosis in the data processing center, as shown in Figure 1. Here, four problems can be included in WSN node fault diagnosis, which can be described as follows.

Problem 1 The extraction process of the data features is formulated. In WSN, the data collected by different sensors have similarities, including time correlation and space correlation. When WSN node fault occurs, the time and space correlation features will be changed. The raw data collected by the sensors therefore need to be analysed, and data features that are time-correlated or space-correlated from the data are extracted as input attributes to the model. The extraction process is described as Eq. (1):

(1) $$X=f\left(\bar{X},\psi \right)$$

where $X=\left\{x_{1},\cdots ,x_{M}\right\}$ denotes the set of attributes used as inputs of the model. M is the number of attributes. f(·) denotes the extraction process of data features. $\bar{X}$ denotes the raw dataset collected by different sensors. ψ denotes the parameter set of the data feature extraction process.

Problem 2 All fault diagnosis results, including global ignorance and local ignorance, are defined. In WSN node fault diagnosis, fault types are used as output of the model. Define Ω as a set of all WSN fault types, which can be described as Eq. (2):

(2) $$\Omega \;=\;\left\{{\text{D}}_{1}\cdots {\text{D}}_{N}\right\}$$

where $D_{i}$ is the ith fault type of WSN node and N represents the number of WSN node fault types. In fault diagnosis of WSN node, local ignorance represents the case where the fault may be any J of all N faults, where J<N. Global ignorance represents the case where the fault may be any one of all N faults. The set of fault types with local ignorance and global ignorance can then be described as Eq. (3):

(3) $$2^{\text{Ω}}=\left\{\emptyset ,D_{1}\text{,}\cdots ,D_{N}\text{,}\left\{D_{1}\text{,}D_{N}\right\}\text{,}\cdots \left\{D_{1}\text{,}\cdots D_{N-1}\right\}\text{,}\text{Ω}\right\}$$

where ∅ is an empty set and $\left\{D_{i},D_{j}\right\}$ represents that WSN node fault diagnosis result may be $D_{i}$ or $D_{j}$, which is used to describe local ignorance. Ω is a complete set, which is used to describe global ignorance. Therefore, the fault diagnosis result of N fault types has $2^{N}$ possibilities.

Problem 3 The fault diagnosis process of WSN node is designed. The fault diagnosis process can be expressed by the following Eq. (4):

(4) $$y=g\left(x_{1},\cdots ,x_{M},\eta \right)$$

where $x_{1},\cdots ,x_{M}$ represents data features extracted in problem 1, i.e., the input attributes of the model. η represents the parameter set of the fault diagnosis process. g(·) indicates the diagnosis process of a fault. y represents the power set of the output of the model defined in problem 2.

Problem 4 The optimization process of the fault diagnosis model is designed. The initial parameters of the model are determined by expert knowledge. It follows the general trend of the belief distribution, but it is not the optimal solution. Therefore, parameters need to be adjusted by optimization algorithm to improve the diagnostic accuracy of the model. The optimization process of the parameters of the model can be described as Eq. (5):

(5) $$\eta_{best}=h\left(\eta_{0},\emptyset \right)$$

where h(⋅) denotes the optimization process of parameters of the model. ∅ is parameter set of the optimization process, and $\eta_{0}$ is the set of parameters of the model initialized by expert knowledge. $\eta_{best}$ is the optimized parameter set.

### 3.2 Construction of the new WSN node fault diagnosis model

To solve the above four problems, a new WSN node fault diagnosis model based on PBRB is proposed. In PBRB, belief rules contain a power set identification framework of output results and input attributes, but this power set framework is the basic probability distribution and not the final result. The final result needs to be derived by a rule fusion algorithm. K belief rules are constructed, and each belief rule can be described as Eq. (6):

(6) $$\begin{array}{l} R_{k}:IF\left(x_{1}\text{ ​}is\text{ ​}A_{1}^{k}\right),\cdots ,\left(x_{M}\text{ ​}is\text{ ​}A_{M}^{k}\right)\\ \;THEN\left\{\left(D_{1},\beta_{1}^{k}\right),\cdots ,\left(D_{2^{N}},\beta_{2^{N}}^{k}\right)\right\},\sum_{n=1}^{2^{N}}\beta_{n}^{k}=1\\ \;WITH\text{ ​}rule\text{ ​}weight\text{ ​}\theta_{k}\text{ ​}and\text{ ​}attribute\text{ ​}weight\text{ ​}\delta_{1},\cdots ,\delta_{M} \end{array}$$

where $R_{k}\left(k=1,\cdots ,K\right)$ denotes the *kth* belief rule in WSN node fault diagnosis model. $A_{1}^{k},\cdots ,A_{M}^{k}$ denotes the value of the reference point of M input attributes of this model, which is defined by experts. $D_{1},\cdots ,D_{2^{N}}$ are the set of fault types in the power set. $\beta_{n}^{k}\left(n=1,\cdots ,2^{N}\right)$ denotes the belief degree of different output results in the power set. $\theta_{k}$ is the weight of the *kth* belief rule, which is used to describe the importance of the rule, and $\delta_{1},\cdots ,\delta_{M}$ are the weights of different attributes, which can reflect the importance of attributes.

**Figure 2:**
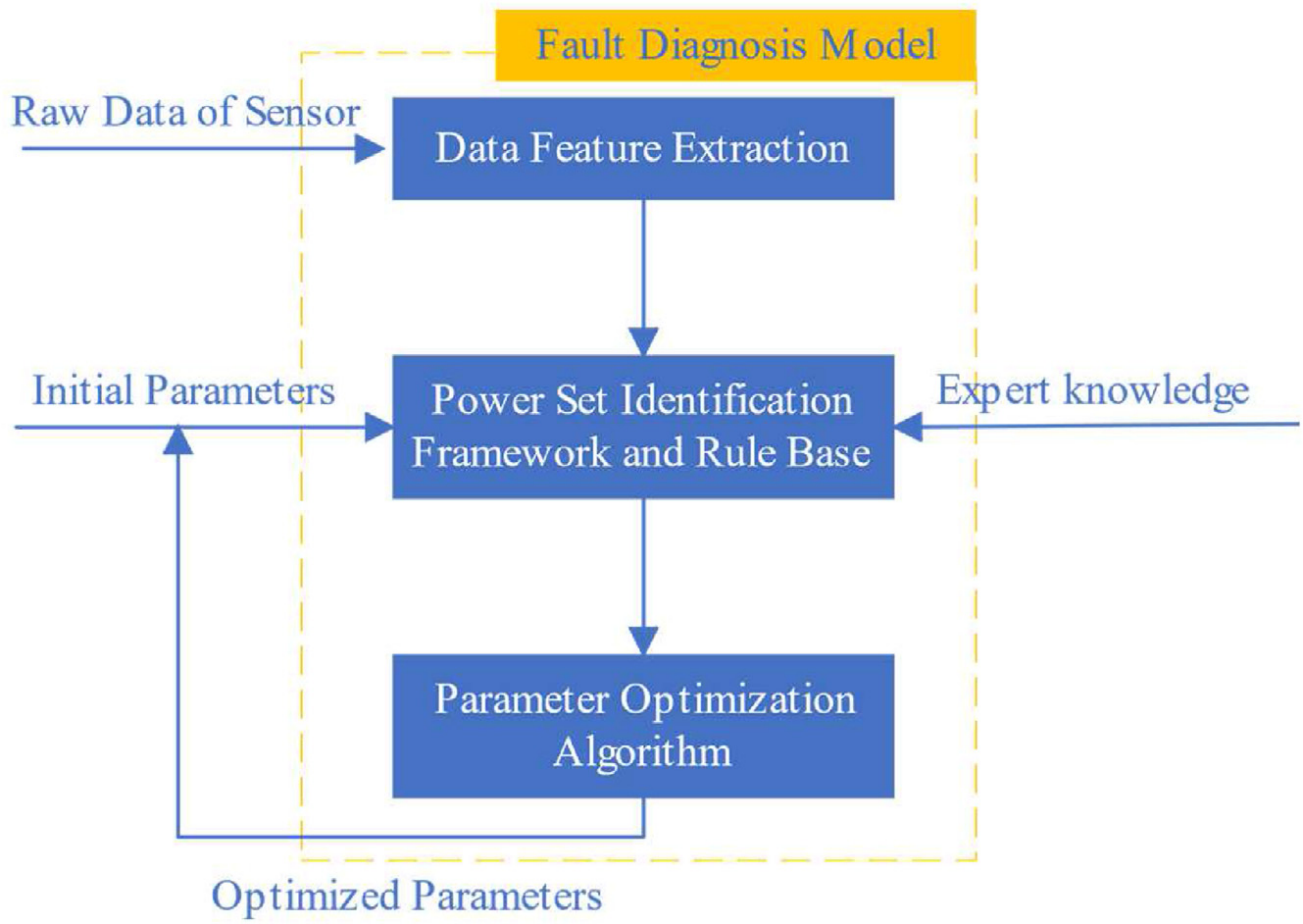
The WSN node fault diagnosis model based on PBRB.

The new WSN node fault diagnosis model based on PBRB comprises the following units. First, a WSN data feature extraction method was designed to extract time-correlated or space-correlated data features to be used as input attributes for the PBRB. Second, the features of the power set identification framework of the PBRB method can be used to represent the local ignorance and global ignorance of the fault diagnosis results of WSN nodes and improve fault diagnosis accuracy. Third, the initial parameters set by experts in PBRB are optimized using an optimization algorithm to further improve the diagnostic accuracy of the model. The fault diagnosis model consisting of the above three components can be represented as Figure 2.

## 4 The WSN node fault diagnosis model based on PBRB

In this section, the modeling process of WSN node fault diagnosis is defined, which includes the basic structure of the model, the reasoning process of the model, and the optimization process of the model.

### 4.1 The basic structure of the model

To effectively describe WSN node fault diagnosis problem in modeling. The fault mechanism and data features of the WSN are analyzed. The basic structure of WSN node fault diagnosis is constructed, which can be described as.

Step 1: The input attributes of the model are constructed. WSN node faults cannot be directly represented by sensor raw data, so WSN node data features are extracted as the input of the model.

**Figure 3:**
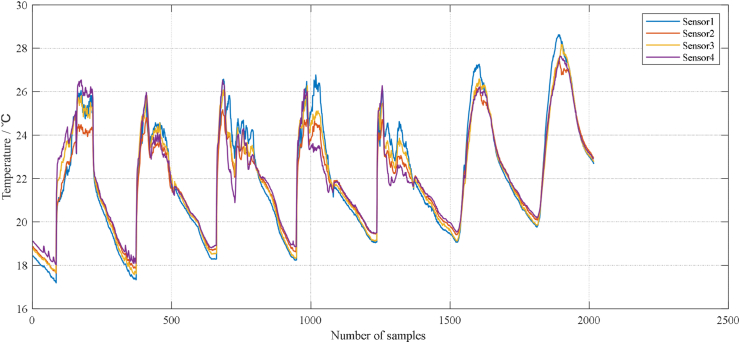
Temperature data of adjacent sensors.

A WSN is a distributed data collection network. There are many sensors that have the same function to collect information on detected objects. As shown in Figure 3, there are certain similarity characteristics in space and time for the information of these sensors, which are shown as follows. The first is time correlation. The overall trend of the object being tested is consistent over a period of time, and therefore, the data collected by the sensor have a similar trend over time. For example. If a WSN is used to monitor temperature changes in an area and the overall temperature in the area is on an upwards trend over a period of time, then the data collected by the sensors distributed in the area are all on an upwards trend and have similarity. The second is spatial correlation, where the difference between two monitored points is smaller the closer they are to the object being detected. Therefore, sensors that are distributed closer together have a strong similarity in monitoring data. For example, still monitoring temperature changes in a certain area, when the distance between two sensors is only 1 m, they have almost the same monitoring data.

When a WSN node failure occurs, the data features of time correlation and space correlation are changed. The data features of different types of faults are also different. Therefore, the data features of the time correlation and space correlation of WSN node are selected as the input of the model.

Trend correlation is an expression of time correlation. It indicates the degree of similarity in the trend of the collected data over a period. which can be calculated by the following Eq. (7):

(7) $$\chi =\frac{\sum_{k=0}^{T}\left[x_{i}\left(t-k\right)-{\bar{x}}_{i}\left(t\right)\right]\left[x_{j}\left(t-k\right)-{\bar{x}}_{j}\left(t\right)\right]}{\sqrt{\sum_{k=0}^{T}{\left[x_{i}\left(t-k\right)-{\bar{x}}_{i}\left(t\right)\right]}^{2}\sum_{k=0}^{T}{\left[x_{j}\left(t-k\right)-{\bar{x}}_{j}\left(t\right)\right]}^{2}}}$$

where $x_{i}\left(t-k\right)\text{ ​ ​}k=\left(0,\cdots ,T\right)$ denotes the data collected by sensor i at time [t−T,t]. ${\bar{x}}_{i}\left(t\right)$ denotes the average value of the data collected by sensor i from time t−T to time t.

The residual feature is defined to represent space correlation, which can be described as Eq. (8):

(8) $$\hat{\varepsilon }=x_{i}\left(t\right)-\frac{1}{S-1}\sum_{j=1,j\neq i}^{S}x_{j}\left(t\right)$$

where $x_{i}\left(t\right)$ denotes the data collected by sensor i at time t. $1/\left(S-1\right)\sum_{j=1,j\neq i}^{S}x_{j}\left(t\right)$ denotes the average data of other sensors collected at time t. S denotes the number of sensors.

More precisely, trend correlation and residual features are extracted as the input attributes of the fault diagnosis model proposed in this paper.

Step 2: The output of the model is constructed. In WSN node fault diagnosis model, fault types are used as the output of the model. WSN node fault types are classified by offset fault, high noise fault, outlier fault and fixed value fault [29]. All these faults can be described as Eq. (9):

(9) $$\Omega =\left\{D_{1},D_{2},D_{3},D_{4}\right\}$$

where $D_{1}$ is the offset fault, $D_{2}$ is the high noise fault, $D_{3}$ is the outlier fault, and $D_{4}$ is the fixed value fault.

The data features between fault types are sometimes similar, making it difficult for the model to distinguish between specific fault types and generate local ignorance and global ignorance. To represent local ignorance and global ignorance information more effectively, a frame of discernment with a power set is defined, which can be described as Eq. (10):

(10) $$2^{\Omega }=\left\{\begin{array}{l} \emptyset ,D_{1},D_{2},D_{3},D_{4},\\ \left\{D_{1},D_{2}\right\},\left\{D_{1},D_{3}\right\},\left\{D_{1},D_{4}\right\},\\ \left\{D_{2},D_{3}\right\},\left\{D_{2},D_{4}\right\},\left\{D_{3},D_{4}\right\},\\ \left\{D_{1},D_{2},D_{3}\right\},\left\{D_{1},D_{2},D_{4}\right\},\\ \left\{D_{1},D_{3},D_{4}\right\},\left\{D_{2},D_{3},D_{4}\right\},\Omega \end{array}\right\}$$

where ∅ means that the current state may not be any of the already defined fault types. $D_{i}\text{ ​}\left(i=1,2,3,4\right)$ means that the current fault is $D_{i}$. $\left\{D_{i},D_{j}\right\}\text{ ​}i,j=1,2,3,4\text{ ​}i\neq j$ means that the current state may be fault $D_{i}$ or fault $D_{j}$. $\left\{D_{i},D_{j},D_{k}\right\}$ and $\left\{D_{i},D_{j}\right\}$ have similar meanings. Ω represents that the current fault may be of any of the types already defined.

Step 3: The belief rules of the model are defined as Eq. (11):

(11) $$\begin{array}{l} R_{k}:IF\text{ ​}x_{1}\text{ ​}is\text{ ​}A_{1}^{k}\text{ ​}and\text{ ​}x_{2}\text{ ​}is\text{ ​}A_{2}^{k}\\ \;THEN\text{ ​}\left\{\begin{array}{l} \left(\emptyset ,\beta_{1}^{k}\right),\left(D_{1},\beta_{2}^{k}\right),\left(D_{2},\beta_{3}^{k}\right),\left(D_{3},\beta_{4}^{k}\right),\\ \left(D_{4},\beta_{5}^{k}\right),\left(\left\{D_{1},D_{2}\right\},\beta_{6}^{k}\right),\left(\left\{D_{1},D_{3}\right\},\beta_{7}^{k}\right),\\ \left(\left\{D_{1},D_{4}\right\},\beta_{8}^{k}\right),\left(\left\{D_{2},D_{3}\right\},\beta_{9}^{k}\right),\\ \left(\left\{D_{2},D_{4}\right\},\beta_{10}^{k}\right),\left(\left\{D_{3},D_{4}\right\},\beta_{11}^{k}\right),\\ \left(\left\{D_{1},D_{2},D_{3}\right\},\beta_{12}^{k}\right),\left(\left\{D_{1},D_{2},D_{4}\right\},\beta_{13}^{k}\right),\\ \left(\left\{D_{1},D_{3},D_{4}\right\},\beta_{14}^{k}\right),\left(\left\{D_{2},D_{3},D_{4}\right\},\beta_{15}^{k}\right),\\ \left(\Omega ,\beta_{16}^{k}\right) \end{array}\right\}\\ \;WITH\text{ ​}rule\text{ ​}weight\text{ ​}\theta_{k}\text{ ​}and\text{ ​}attribute\text{ ​}weight\text{ ​}\delta_{1},\delta_{2} \end{array}$$

where $x_{1}$ and $x_{2}$ represent the input attributes of the model. The WSN node fault diagnosis model in this paper represents trend correlation and residual features. $A_{1}^{k}$ and $A_{2}^{k}$ represent the reference points of the input attribute, which are set by expert knowledge. $\beta_{1}^{k},\cdots ,\beta_{16}^{k}\left(k=1,\cdots ,L\right)$ represents the rule basic probability distribution, and $\sum_{i=1}^{16}\beta_{i}^{k}=1$. ∅ $D_{i}\text{ ​}\left(i=1,2,3,4\right)$$\left\{D_{i},D_{j}\right\}\text{ ​}i,j=1,2,3,4\text{ ​}i\neq j$, and $\left\{D_{i},D_{j},D_{k}\right\}$ have the same meaning as in Eq. (10). $\theta_{k},k=1,2,\cdots ,L$ represents the rule weight of each rule, and $\delta_{1},\delta_{2}$ represents the input attribute weight. Among these parameters, the input attribute reference point, basic probability distribution, rule weight and input attribute weight have expert knowledge to be initialized.

### 4.2 The Inference process of the model

**Figure 4:**
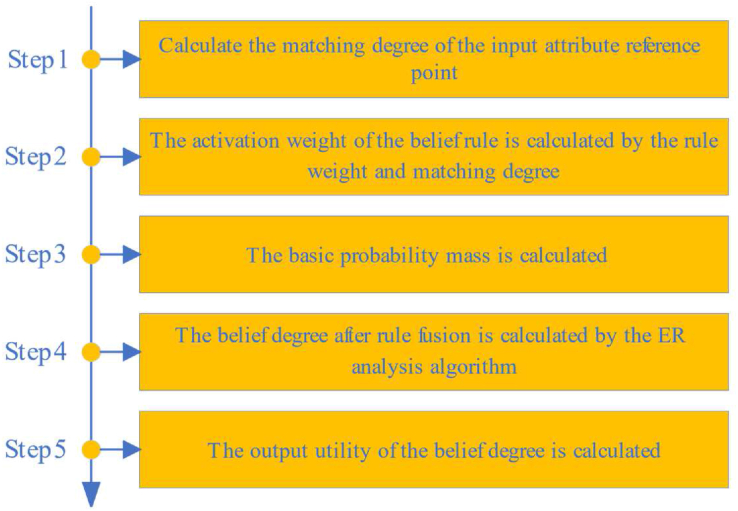
The Inference process of WSN node fault diagnose.

The inference process of WSN node fault diagnosis is designed in this part, as shown in Figure 4. The specific reasoning process is described as follows.

Step 1: The input and reference values of the attributes are used to calculate the matching degree of the reference point of the corresponding attributes. First, assume that attribute ith has m reference values of $\left[A_{i}^{1},\cdots ,A_{i}^{k},A_{i}^{k+1},\cdots ,A_{i}^{m}\right]$, and they are sorted in an increasing order. Then, the matching of reference values at positions k and k+1 can be calculated by the following Eq. (12):

(12) $$a_{i}^{j}=\left\{ \begin{array}{l} \frac{A_{i}^{k+1}-x_{i}}{A_{i}^{k+1}-A_{i}^{k}},\text{ ​ ​ ​ ​}j=k,A_{i}^{k}\leq x_{i}\leq A_{i}^{k+1}\\ \frac{x_{i}-A_{i}^{k}}{A_{i}^{k+1}-A_{i}^{k}},\text{ ​ ​ ​ ​}j=k+1\\ 0,\;\text{ ​}j=1,2,\cdots ,m,\;\text{ ​}j\neq k,\;\text{and}\;j\neq k+1 \end{array}\right.$$

where $a_{i}^{j}$ denotes the matching degree with the jth reference value of the ith attribute. $x_{i}$ is the value of the ith input attribute. $A_{i}^{k}$ and $A_{i}^{k+1}$ represent the two adjacent reference values. If $x_{i}$ is between $\left[A_{i}^{k},A_{i}^{k+1}\right]$, the matching degree of $x_{i}$ for $A_{i}^{k}$ and $A_{i}^{k+1}$ is calculated; otherwise, the matching degree of other reference values is 0.

Step 2: The activation weight of the belief rule is calculated by the rule weight and matching degree, and the process can be described as Eq. (13):

(13) $$\omega^{k}=\frac{\theta_{k}\prod_{i=1}^{M}{\left(a_{i}^{k}\right)}^{\delta_{i}}}{\sum_{j=l}^{K}\theta_{j}\prod_{i=1}^{M}{\left(a_{i}^{j}\right)}^{\delta_{i}}}$$

where $\omega^{k}$ denotes the activation weight of the kth belief rule. $\theta_{j}\text{ ​ ​}\left(j=1,2,\cdots ,K\right)$ denotes rule weight of the jth rule, and K is the total number of rules. $a_{i}^{j}$ denotes the matching degree of the ith attribute on the corresponding reference value in the jth rule. $\delta_{i}$ represents attribute weight of the ith attribute. When $\omega^{k}\neq 0$, the current rule is activated.

Step 3: The basic probability mass is calculated, which can be described as Eqs. (14) and (15):

(14) $$m_{n}^{k}=\omega^{k}\beta_{n}^{k}$$

(15) $${\bar{m}}_{2^{\Omega }}^{k}=1-\omega^{k}$$

where $\omega_{k}$ represents the activation weight of the kth rule. $\beta_{n}^{k}$ represents the belief degree of the nth outcome in the discriminative framework for the kth rule. $m_{n}^{k}$ denotes the basic probability mass of the kth(k=1,⋯,K) belief rule for the $nth\left(n=1,\cdots ,2^{N}\right)$ fault state. ${\bar{m}}_{2^{\Omega }}^{k}$ denotes the basic probability mass that is not assigned to the fault state in the kth belief rule.

Step 4: The belief degree after rule fusion is calculated by the ER analysis algorithm, which can be described as Eqs. (16) and (17):

(16) $$\kappa =\frac{l}{\sum_{n=1}^{2^{N}}\prod_{k=1}^{K}\left(m_{n}^{k}+{\bar{m}}_{2^{\Omega }}^{k}\right)-\left(2^{N}-1\right)\prod_{k=1}^{K}{\bar{m}}_{2^{\Omega }}^{k}}$$

(17) $$\beta_{n}=\frac{\kappa \times \left[\prod_{k=1}^{K}\left(m_{n}^{k}+{\bar{m}}_{2^{\Omega }}^{k}\right)-\prod_{k=l}^{K}{\bar{m}}_{2^{\Omega }}^{k}\right]}{l-\kappa \times \left[\prod_{k=l}^{K}{\bar{m}}_{2^{\Omega }}^{k}\right]}$$

where $\beta_{n}\left(n=1,\cdots ,2^{N}\right)$ represents the confidence of the belief degree result in the power set identification framework. N denotes the number of fault types, and K denotes the number of rules. $m_{n}^{k}$ and ${\bar{m}}_{2^{\Omega }}^{k}$ are calculated by Eqs. (14) and (15).

Step 5: The output utility of the belief degree is calculated as Eq. (18):

(18) $$y=\sum_{n=1}^{2^{N}}D_{n}\beta_{n}$$

where $D_{n},\text{ ​ ​}n=1,2,\cdots ,2^{N}$ represents the $2^{N}$ results in the power set identification framework. $\beta_{n},\text{ ​ ​}n=1,2,\cdots ,2^{N}$ represents the belief degree of $2^{N}$ resulting in the power set identification framework. N denotes the number of fault types. For example, there exists a discriminatory framework {(1,0.2),(2,0.3),(3,0.5)}. Then, the final output utility y is 1×0.2+2×0.3+3×0.5=2.3. The calculation result in this case is closest to 2, so the classification result is 2.

### 4.3 The optimization process of the model

**Figure 5:**
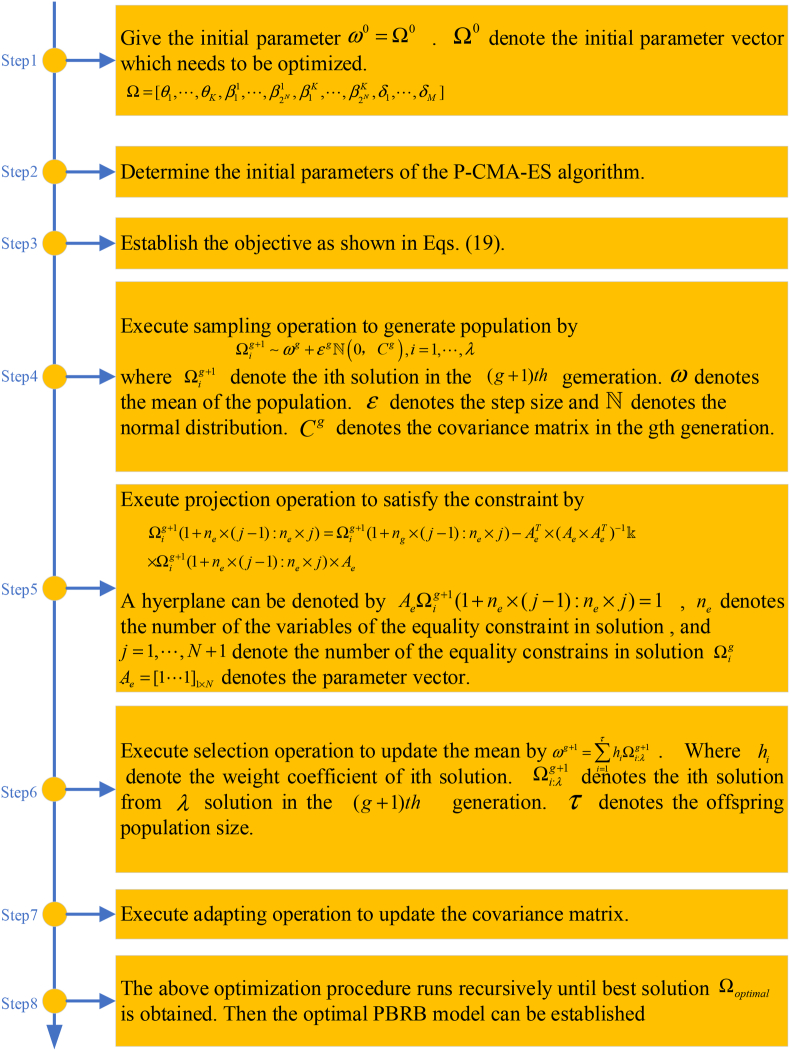
Flowchart of the P-CMA-ES optimization algorithm.

The initial parameters of WSN node fault diagnosis model are constructed based on expert knowledge. However, there are two problems with this. First, the extracted data features are similar in some cases. Second, as the number of attributes increases, setting the initial parameters becomes more difficult. The initial parameters set by expert knowledge are consistent with the working mechanism of the WSN to some extent, but they are not optimal. Therefore, the model needs to be trained by the data to obtain more accurate model parameters. The optimization objective function can be expressed in the following Eq. (19):

(19) $$\begin{array}{l} \;\min MSE\left(\eta \right)\\ s.t.\\ \;\sum_{n=1}^{2^{N}}\beta_{n}^{k}=1\\ \;0\leq \beta_{n}^{k}\leq 1,\;k=1,\cdots ,K,\;n=1,\cdots ,2^{N}\\ \;0\leq \theta_{k}\leq 1,\;k=1,\cdots ,K\\ \;0\leq \delta_{m}\leq 1,\;m=1,\cdots ,M \end{array}$$

where $\eta =\left[\theta_{1},\cdots ,\theta_{K},\beta_{1}^{1},\cdots ,\beta_{2^{N}}^{1},\beta_{1}^{K},\cdots ,\beta_{2^{N}}^{K},\delta_{1},\cdots ,\delta_{M}\right]$ denotes the parameter set of the fault diagnosis model. The mean square error (MSE) is used as the objective function of the optimization algorithm and is denoted using MSE(·). The objective function can be expressed as Eq. (20):

(20) $$MSE\left(\eta \right)=\frac{1}{NUM}\sum_{i=1}^{NUM}\left(y^{i}-y_{excepted}^{i}\right)$$

where NUM represents the number of training samples. $y^{i}$ is the actual output of the ith training sample in WSN node fault diagnosis model, and $y_{\exp\;ected}^{i}$ is the expected output of the ith training sample. The projection covariance matrix adaptation evolution strategy (P-CMA-ES) algorithm is selected to optimize the model parameters, as shown in Figure 5.

### 4.4 Process of the model

Through the above analysis, the modelling process of WSN node fault diagnosis is designed. This can be described as follows.

Step 1: The correlation features of the sensor data are extracted and used as the input attributes of the diagnosis model.

Step 2: The WSN node fault diagnosis model based on the PBRB is constructed by expert knowledge.

Step 3: The reasoning process of the fault diagnosis model is designed based on the ER parsing algorithm.

Step 4: The P-CMA-ES algorithm was chosen as the optimization algorithm for the initial parameters of the diagnostic model.

## 5 Case study

In this section, a case study is designed to verify the effectiveness of the proposed method in this paper, including problem formulation, construction, training and testing of the model.

### 5.1 Problem formulation

**Figure 6:**
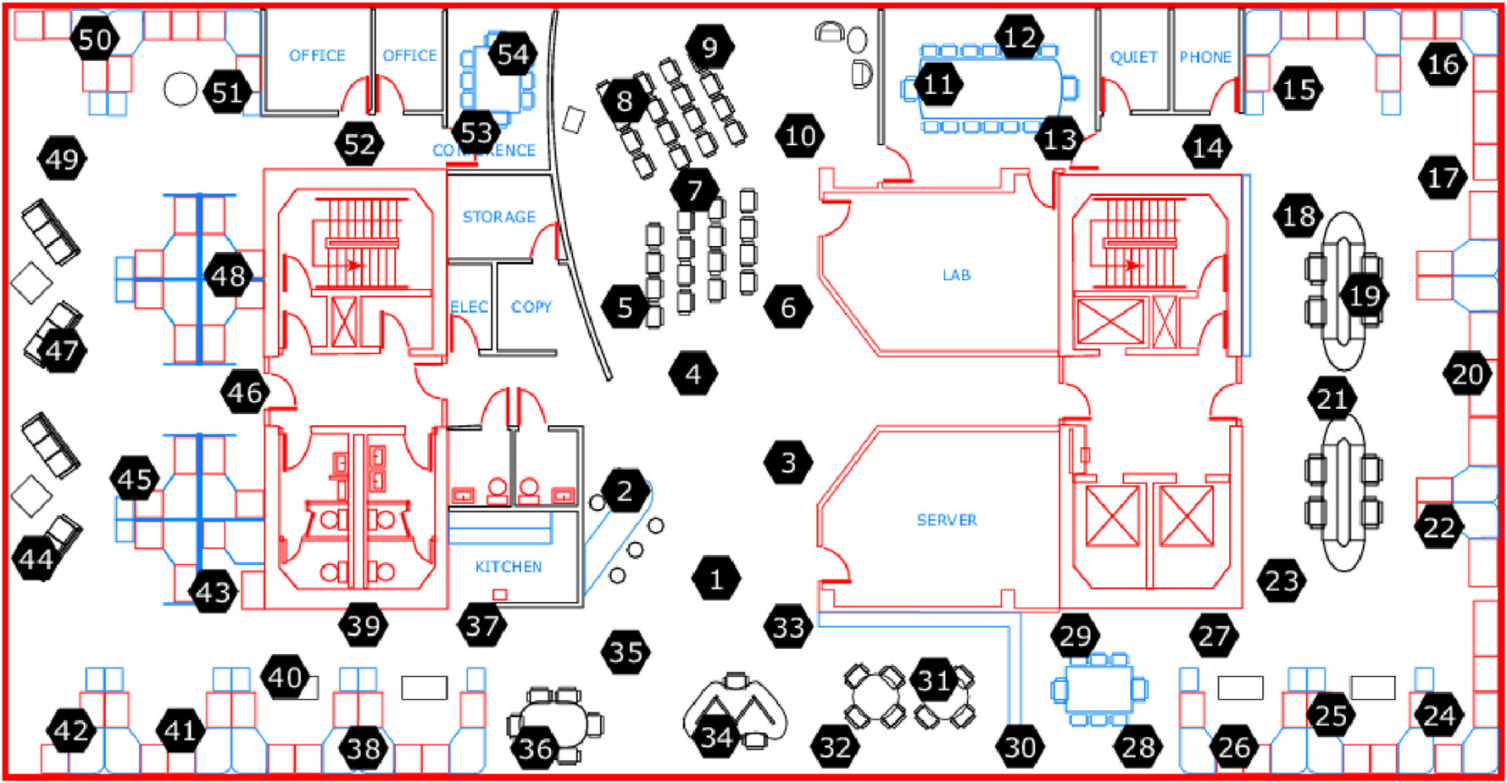
Wireless Sensor Network distribution map in Intel Berkeley Research Lab.

In this case study, WSN datasets collected and published by Intel Berkeley Research Labs were selected. This dataset contains information about data collected from 54 sensors deployed in the Intel Berkeley Research lab between February 28th and April 5th, 2004. The data collected by each sensor node include temperature, humidity, light, and voltage. The sensor is arranged in the laboratory according to Figure 6. Sensors 1, 2, 3, and 4 are selected as the data sources for this article based on the sensor installation location.

Step 2: Raw data is processed on demand. By analyzing the characteristics of the data set, it is found that the sensor has data loss phenomenon in a period of time. The data volume and time of sensors 1 to 4 are inconsistent. Therefore, on the basis of the source data, the method of average value is adopted to make up the missing data. Finally, data from sensors 1 to 4 were processed every 5 min from March 1 to March 7, resulting in a total of 2016 data from each sensor.

**Figure 7:**
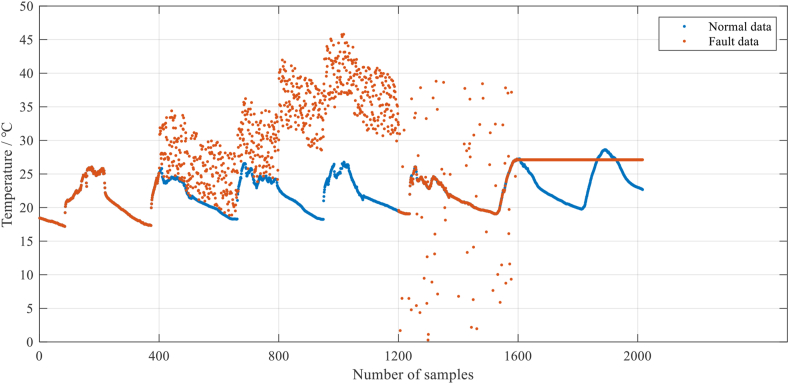
Simulated fault data on sensor 1.

Step 3: Sensor failure data are simulated on senser 1. The simulation method is shown in Table 1. The effect after simulating fault data in sensor 1 is shown in Figure 7, where 1–399 are normal data and 400–799 are offset fault data. 800–1199 are high noise fault data, 1200–1599 are outlier fault data and 1600–2016 are fix value fault data.

**Table 1:** Simulation method of different type of fault.

Fault type	Simulation method
Offset fault	Randomly superimpose a random number between 0 and 10 on the sample 400–799.
High noise fault	Randomly superimpose a random number between 10 and 20 on the sample 800–1199.
Outlier fault	Randomly draw 10% of discrete data samples from samples 1200–1599 and replace them with random numbers between 0 and 40.
Fix value fault	Change the value of sample 1600–2016 to the value of sample 1599

### 5.2 Construction of the model

**Figure 8:**
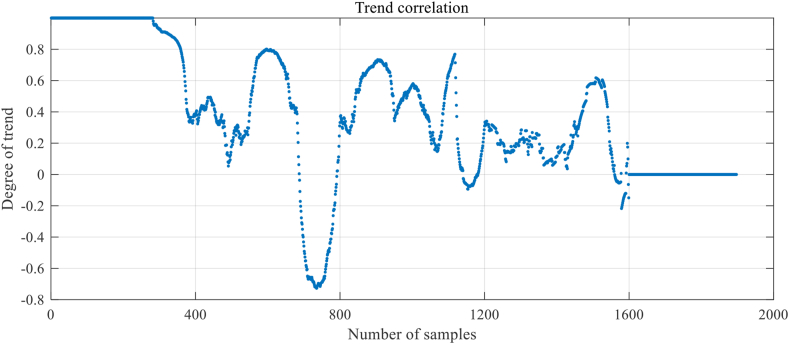
Results of trend correlation.

**Figure 9:**
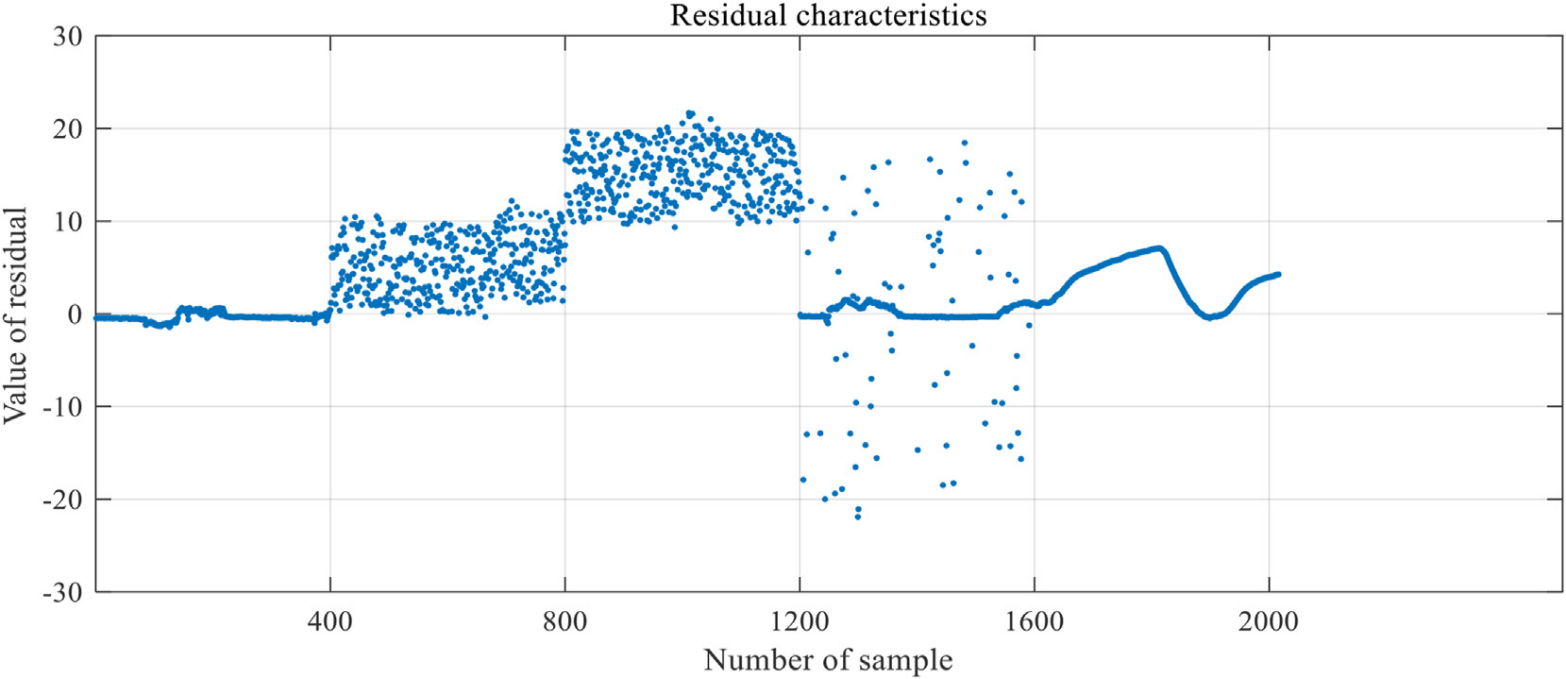
Results of residual characteristics.

**Figure 10:**
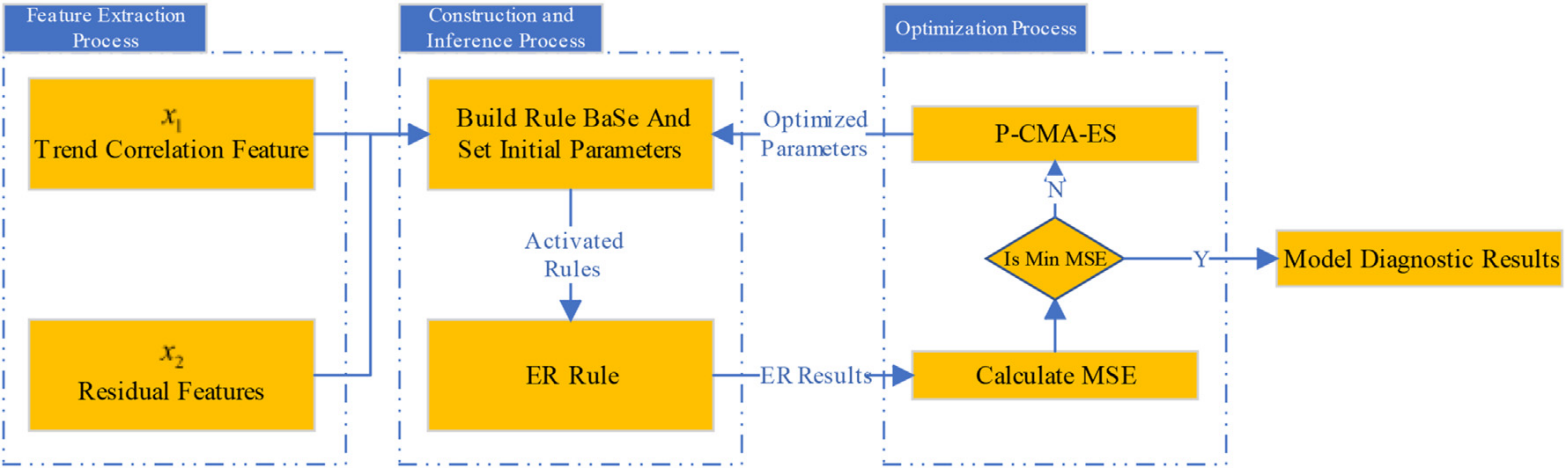
Simple structure of model.

Step 1: Extracting data features include trend correlation and residual feature. To build a fault diagnosis model for WSN nodes, data features need to be extracted from the raw sensor data first. In this paper, the trend correlation is calculated by the method shown in Eq. (7) and represented by $x_{1}$. The residual feature is calculated by the method shown in Eq. (8) and represented by $x_{2}$. The calculation results of the trend correlation and residual feature are shown in Figure 8 and Figure 9, respectively. A schematic diagram of the model structure is shown in Figure 10.

Step 2: The reference point and reference value of the input attributes are defined. By analyzing the data features and graphs of the input attributes $x_{1}$ and $x_{2}$, the reference points of the two input attributes can be determined. First, there are 7 reference points for $x_{1}$, which are very low (VL), relatively low (RL), low (L), medium (M), high (H), relatively high (RH), and very high (VH) and can be described as Eq. (21). The reference value corresponding to the reference point is shown in Table 2. Second, there are 7 reference points for $x_{2}$, which are very low (VL), relatively low (RL), low (L), medium (M), high (H), relatively high (RH), and very high (VH), as described in Eq. (22). The reference value corresponding to the reference point is shown in Table 3. Finally, 5 reference points are set for the output result of the model, which are normal (N), offset fault (OSF), high noise fault (HNF), outlier fault (OF), and fix value fault (FVF), which can be described as Eq. (23). The reference value is shown in Table 4.

(21) $$x_{1}=\left\{VL,RL,L,M,H,RH,VH\right\}$$

(22) $$x_{2}=\left\{VL,RL,L,M,H,RH,VH\right\}$$

(23) y={N,OSF,HNF,OF,FVF}

**Table 2:** Reference point and reference value of trend correlation.

Reference point	VL	RL	L	M	H	RH	VH
Reference value	-1.1	0	0.2	0.4	0.6	0.8	1.1

**Table 3:** Reference point and reference value of residual characteristics.

Reference point	VL	RL	L	M	H	RH	VH
Reference value	-23	-10	0	5	10	15	23

**Table 4:** Reference point and reference value of model output.

Reference point	N	OSF	HNF	OF	FVF
Reference value	0	1	2	3	4

Following the process of model construction introduced in Section 4.1, the power set framework needs to be constructed based on the set of fault types y. Following the process of model construction introduced in Section 4.1, the power set framework needs to be constructed based on the set of fault types Y. However, observing the images of the data features, it is found that only adjacent faults in the defined faults have local ignorance. Therefore, the local ignorance and global ignorance that do not exist in the power set framework can be reduced. Eventually, the discriminative framework $y^{\prime }$ of the model is obtained as Eq. (24).

(24) $$\begin{array}{l} y^{\prime }=\{N,\{N,OSF\},OSF,\{OSF,HNF\},HNF,\\ \{HNF,OF\},OF,\{OF,FVF\},FVF\} \end{array}$$

Step 3: The initial belief rule base consisting of 49 rules is constructed by the data features extracted in Step 1 and the reference point and reference value determined in Step 2.

### 5.3 Training and testing of the model

In this part, the model constructed in Section 5.2 will be trained. After the model is trained, the test data will be used to verify the accuracy and effectiveness of the model.

Step 1: The training data and iteration number are determined. In Step 3 of Part 1 of this section, different types of fault data on sensor 1 are simulated and data features are extracted from the simulated data. The 1897 extracted data features were randomly divided into six groups of 8:2, 7:3, 6:4, 5:5, 4:6, and 3:7 according to the ratio of training and test sets commonly used in machine learning.

Step 2: Three evaluation indexes are defined in this step to verify the effectiveness of the model. The first index is overall accuracy. which can be described as Eq. (25):

(25) $$Overall\_acc=\frac{TN}{all}\times 100$$

where TN denotes the number of samples correctly diagnosed. all denotes the total number of samples. The second index is fault diagnosis accuracy, which can be described as Eq. (26):

(26) $$Fault\_acc=\frac{FN^{\prime }}{FN}\times 100$$

where FN denotes the number of fault samples and $FN^{\prime }$ denotes the number of correctly diagnosed fault samples. The last index is the fault detection rate, which can be described as Eq. (27):

(27) $$Check\_rate=\frac{FN"}{FN}\times 100$$

where FN denotes the number of fault samples and FN" denotes the number of samples in which the diagnosis result is a fault sample and the source data is also a fault sample.

**Figure 11:**
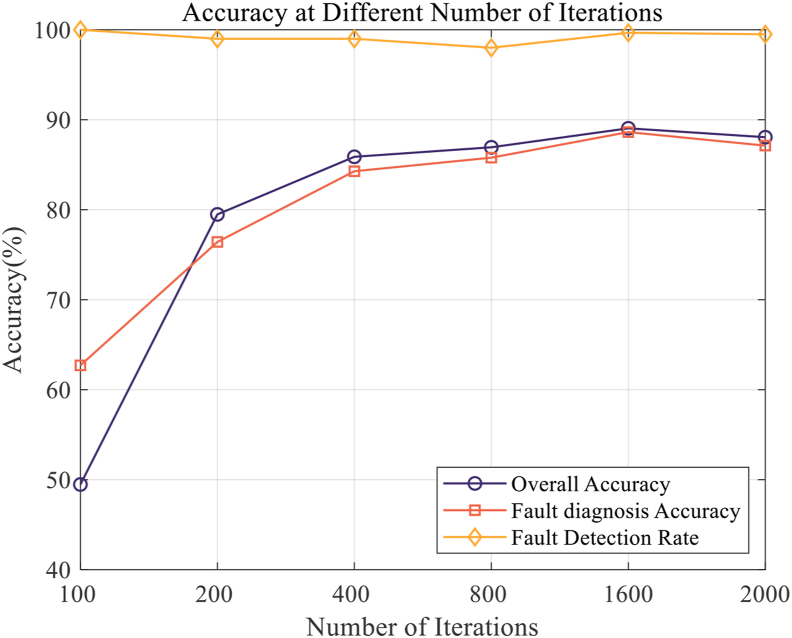
Variation trend of model accuracy with the number of iterations.

Step 3: The number of model iterations is determined, and the model is trained. In the process of model training, the iteration times of model training are set as 100, 200, 400, 800, 1600 and 2000. By comparing the performance indicators defined in Step 2 under different iterations, it is found that the accuracy of the model improves with the increase of the number of training iterations. The improvement in model accuracy is shown in Figure 11. Finally, the number of model iterations is determined to be 1600.

### 5.4 Optimization algorithm comparison

**Figure 12:**
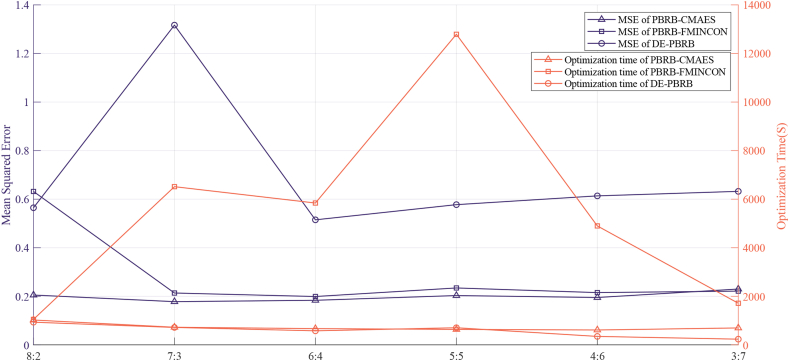
Comparison of optimization algorithm results.

In this part, the parameters of the model are optimized. The rule weights, attribute weights, and belief degree parameters are initially set by the experts, but are not yet the optimal set of parameters for fault diagnosis. Therefore, the set of parameters needs to be adjusted by an optimization algorithm to obtain better diagnostic results. Common optimization algorithms for BRB methods are the differential evolution algorithm (DE) [30], the FMINCON function of the MATLAB optimization toolbox [31], and the P-CMA-ES method [32, 33]. To choose the most suitable optimization algorithm for this paper, each of the three optimization algorithms was tested using six different scales of data defined in the first step of Part C. The test results are shown in Figure 12. First, the comparison of MSE values shows that the DE algorithm has the largest MSE value and the worst optimization effect. The MSE values of the FMINCON and P-CMA-ES methods are very close, and the P-CMA-ES method is slightly better than the FMINCON method. Second, in terms of the length of time for optimization, the DE algorithm and the P-CMA-ES method take approximately the same amount of time. However, considering the large MSE of the DE method, finally, the P-CMA-ES method was chosen as the optimization method for the model parameters.

After the optimization algorithm is determined, the initial parameters of the model are optimized, and the optimized rule weights and belief degree are shown in Table 5. Where the No column of the table indicates the number of the rule, and there are 49 rules in the current model. The second column represents the weight of the current rule. $x_{1}$ in the Attribute column represents the reference point for the trend correlation, and $x_{2}$ represents the reference point for the residual characteristic.

**Table 5:** Optimized parameters of model.

No.	Rule Weight	Attributes		Belief Degree Distribution of the Discriminative Framework in the Rule
		x1	x2	{N, {N, OSF}, OSF, {OSF, HNF}, HNF, {HNF, OF}, OF,{OF, FVF}, FVF}
1	0.399529974	VL	VL	{0.11545, 0.020355, 0.15021, 0.0073873, 0.2955, 0.088801, 0.2482, 0.055848, 0.018236}
2	0.884038038	VL	RL	{0.074501, 0.03547, 0.308, 0.010765, 0.21753, 0.010129, 0.087667, 0.12902, 0.12691}
3	0.576651296	VL	L	{0.0023282, 0.0037552, 0.0075553, 0.0040417, 0.0078722, 0.25359, 0.0064325, 0.064026, 0.6504}
4	0.903089289	VL	M	{0.44123, 0.12672, 0.15136, 0.054862, 0.068459, 0.011103, 0.10221, 0.042993, 0.0010561}
5	0.304424816	VL	H	{0.33713, 0.37554, 0.01902, 0.013802, 0.008199, 0.097889, 0.023963, 0.06298, 0.061469}
6	0.034714151	VL	RH	{0.12604, 0.32635, 0.019356, 0.018511, 0.25233, 0.0080164, 0.18146, 0.022055, 0.045887}
7	0.952804018	VL	VH	{0.069273, 0.14017, 0.09287, 0.085504, 0.26369, 0.017867, 0.2099, 0.010557, 0.11017}
8	0.3237199	RL	VL	{0.10123, 0.044336, 0.24541, 0.12796, 0.094825, 0.057874, 0.11255, 0.17972, 0.036097}
9	0.569802694	RL	RL	{0.00079271, 0.0020787, 0.0050308, 0.00598, 0.0054318, 0.0071128, 0.010104, 0.33297, 0.6305}
10	0.000594206	RL	L	{0.065542, 0.01169, 0.098833, 0.0062164, 0.098245, 0.04041, 0.25554, 0.3208, 0.10272}
11	0.032400634	RL	M	{0, 0, 0, 0.000267, 0.0010848, 0, 0.0030628, 0.0021301, 0.99364}
12	0.001569747	RL	H	{0.33749, 0.046717, 0.015232, 0.30554, 0.15658, 0.0010073, 0.049411, 0.056738, 0.031285}
13	0.349087936	RL	RH	{0.14313, 0.0086841, 0.15991, 0.011042, 0.31032, 0.10989, 0.13681, 0.0068827, 0.11334}
14	0.009531977	RL	VH	{0.029807, 0.085941, 0.013389, 0.15317, 0.19785, 0.31303, 0.0078153, 0.19111, 0.0078896}
15	0.060143858	L	VL	{0.050049, 0.12411, 0.010426, 0.29478, 0.12299, 0.076335, 0.20129, 0.096598, 0.023434}
16	1	L	RL	{0.0052607, 0.0025745, 0.083712, 0.097321, 0.12056, 0.0045116, 0.0067235, 0.27887, 0.40046}
17	0.142667231	L	L	{0.014407, 0.11414, 0.0016368, 0.11815, 0.012236, 0.060193, 0.23947, 0.36557, 0.074203}
18	0.081354952	L	M	{0.13157, 0.052286, 0.0077227, 0.01513, 0.020888, 0.3866, 0.20403, 0.0034161, 0.17836}
19	0.029183296	L	H	{0.23743, 0.64169, 0.037147, 0.049445, 0.018404, 0.0027982, 0.0025077, 0.0088973, 0.0016894}
20	0.788192356	L	RH	{0.045688, 0.14098, 0.013851, 0.26247, 0.076148, 0.026352, 0.22905, 0.022671, 0.18279}
21	0.644560024	L	VH	{0.21415, 0.062333, 0.070602, 0.088775, 0.025262, 0.27236, 0.091476, 0.013664, 0.16138}
22	0.615972989	M	VL	{0.039148, 0.0107, 0.0080824, 0.016983, 0.11598, 0.051009, 0.335, 0.23488, 0.18822}
23	0.014966941	M	RL	{0.090879, 0.15404, 0.10957, 0.040033, 0.011336, 0.4126, 0.014906, 0.15882, 0.0078213}
24	0.114503188	M	L	{0.30202, 0.12944, 0.24657, 0.098925, 0.11354, 0.043025, 0.014214, 0.0063892, 0.045876}
25	0.398891895	M	M	{0.5326, 0.027558, 0.071872, 0.045141, 0.19132, 0.0020263, 0.027525, 0.039554, 0.062406}
26	7.61169E-05	M	H	{0.2106, 0.27317, 0.023461, 0.04197, 0.021375, 0.0028051, 0.064145, 0.1319, 0.23058}
27	0.889461496	M	RH	{0.12443, 0.016554, 0.081213, 0.12473, 0.17322, 0.20419, 0.20283, 0.01781, 0.05501}
28	0.105470958	M	VH	{0.12453, 0.20933, 0.0069261, 0.071924, 0.052146, 0.29303, 0.025139, 0.024612, 0.19236}
29	0.77626393	H	VL	{0.067203, 0.18075, 0.16794, 0.062219, 0.13276, 0.1368, 0.12478, 0.051967, 0.075566}
30	0.992519391	H	RL	{0.0030521, 0.30969, 0.00039447, 0.050446, 0.10656, 0.18908, 0.1269, 0.21353, 0.0003362}
31	0.187562322	H	L	{0, 0, 0.002528, 0.0033252, 0.10184, 0.0052884, 0.13939, 0.26073, 0.4874}
32	0.646065683	H	M	{0.40952, 0.31575, 0.0040091, 0.037773, 0.022622, 0.0040326, 0.16204, 0.0038513, 0.040403}
33	0.156381892	H	H	{0.00081054, 0.0094361, 0.24418, 0.26878, 0.033386, 0.070094, 0.042281, 0.20089, 0.13015}
34	0.366103612	H	RH	{0.00050531, 0.062234, 0.31206, 0.131, 0.13815, 0.034392, 0.1529, 0.13598, 0.032775}
35	0.297837179	H	VH	{0.09299, 0.1899, 0.024836, 0.064693, 0.30527, 0.030432, 0.065971, 0.014697, 0.2112}
36	0.708587308	RH	VL	{0.024263, 0.018946, 0.045449, 0.22304, 0.075586, 0.084306, 0.12995, 0.094788, 0.30367}
37	0.204247808	RH	RL	{0.11586, 0.15345, 0.01189, 0.16214, 0.010336, 0.10576, 0.0081615, 0.28551, 0.14689}
38	0.37275653	RH	L	{0.61625, 0.30428, 0.049802, 0.0072397, 0.014736, 0.0029189, 0.0027477, 0, 0.002201}
39	0.996848591	RH	M	{0.34157, 0.024249, 0.29396, 0.058938, 0.20486, 0.032458, 0.016887, 0.015002, 0.012081}
40	0.36579236	RH	H	{0.11419, 0.13033, 0.0094586, 0.15198, 0.10677, 0.080684, 0.27026, 0.10252, 0.033802}
41	0.705622324	RH	RH	{0.19704, 0.12974, 0.01917, 0.21543, 0.010068, 0.0078929, 0.095593, 0.010264, 0.3148}
42	0.322165953	RH	VH	{0.17864, 0.097687, 0.052442, 0.031136, 0.025114, 0.2601, 0.03801, 0.22456, 0.092318}
43	0.023256976	VH	VL	{0.012036, 0.087267, 0.032161, 0.18292, 0.079334, 0.18631, 0.15823, 0.10249, 0.15925}
44	0.349244664	VH	RL	{0.31911, 0.19144, 0.021428, 0.019725, 0.044562, 0.02411, 0.065145, 0.05613, 0.25835}
45	0.986747975	VH	L	{0.99506, 0.0024281, 0.0020914, 0, 0.0024628, 0, 0, 0, 0}
46	0.64769478	VH	M	{0.0016223, 0.063919, 0.3821, 0.061863, 0.056823, 0.25212, 0.03316, 0.021439, 0.12695}
47	0.658565611	VH	H	{0.14947, 0.079303, 0.11347, 0.0073799, 0.057477, 0.20393, 0.15675, 0.15326, 0.07896}
48	0.375137552	VH	RH	{0.033137, 0.24617, 0.0073748, 0.028994, 0.036864, 0.03905, 0.21558, 0.14237, 0.25046}
49	0.842594229	VH	VH	{0.026995, 0.016485, 0.15844, 0.05408, 0.091956, 0.084011, 0.021042, 0.19358, 0.35341}

### 5.5 Comparison with other methods

In this section, the index defined in Section 5.3 is used to verify the effectiveness of the model that we proposed, named the PBRB, compared with some other common fault diagnosis methods for wireless sensor network nodes, including backpropagation neural networks (BPNN), K-nearest neighbor (KNN), Extreme Learning Machine (ELM) and Belief Rule Base without power set (BRB).

First, using the PBRB method proposed in this paper, six fault diagnosis experiments are executed according to the division method of training and test sets defined in the first step of Section 5.3. Each group of experiments was recorded for overall accuracy, fault diagnosis accuracy and fault detection rate. These results are calculated by Eq. (25) (26) and (27) and shown in Table 6.

**Table 6:** Results of model based on PBRB.

Experimental group	8:2	7:3	6:4	5:5	4:6	3:7
Overall accuracy	90.50%	88.58%	88.52%	88.50%	86.73%	87.26%
Fault diagnosis accuracy	90.04%	89.04%	87.38%	89.05%	84.64%	87.13%
Fault detection rate	100.00%	100.00%	99.67%	100.00%	99.43%	100.00%

**Figure 13:**
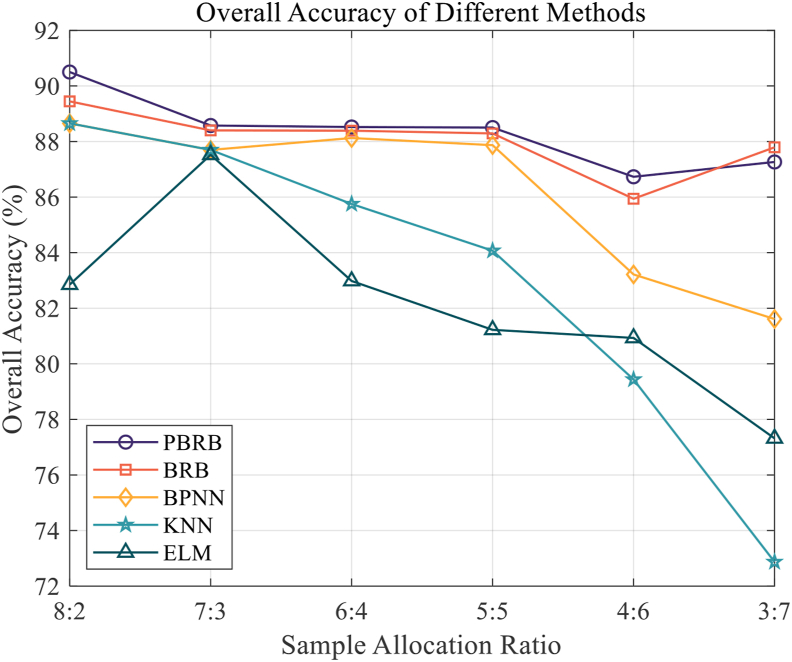
Overall Accuracy of different methods.

**Figure 14:**
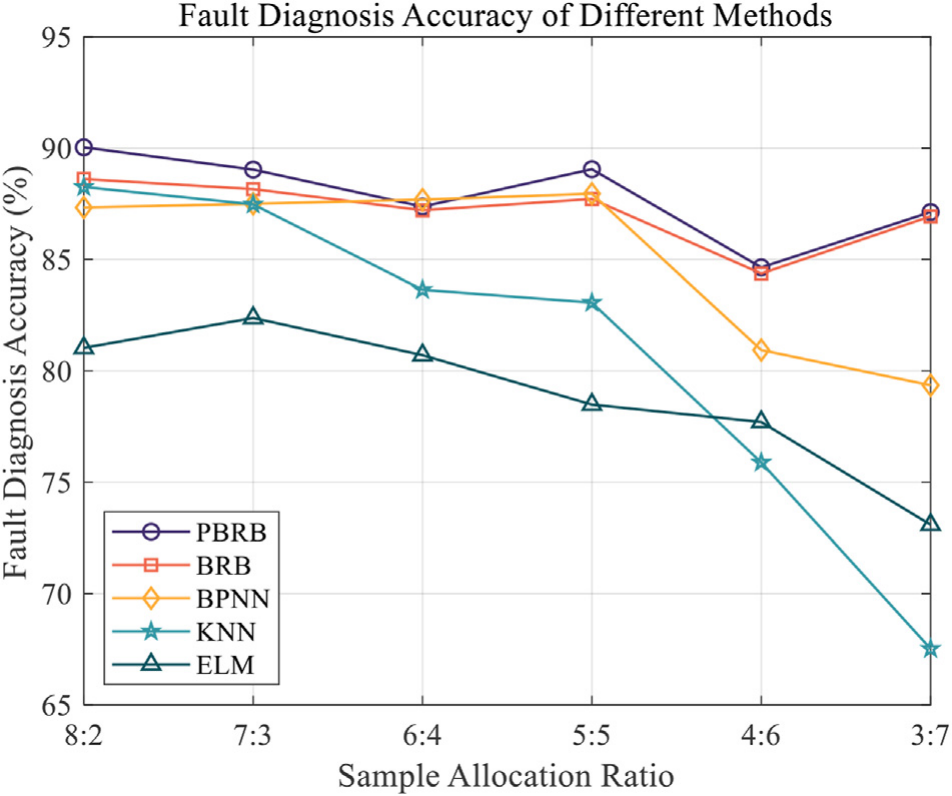
Fault Diagnosis Accuracy of different methods.

**Figure 15:**
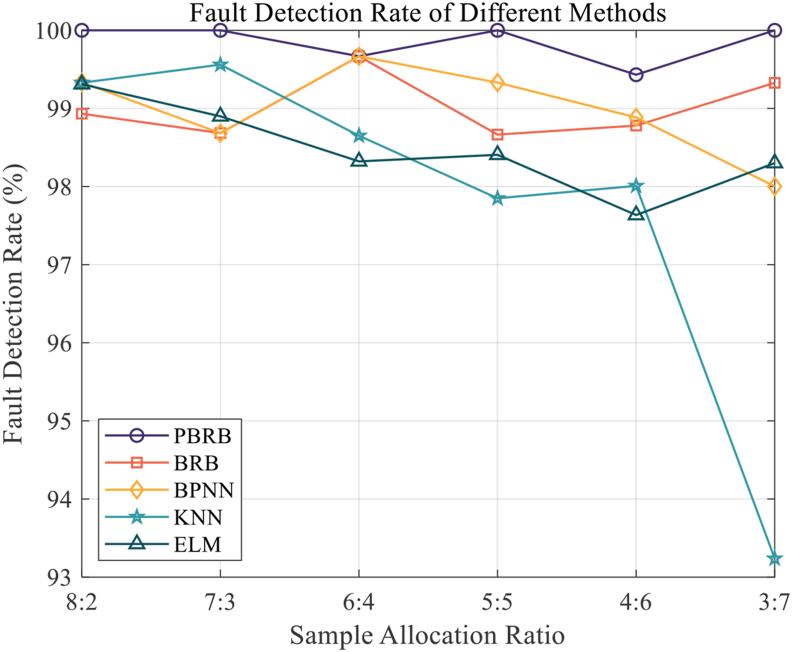
Fault Detection Rate of different methods.

Second, frequently used fault diagnosis methods were chosen as control experiments. where include BPNN, ELM and BRB. The same experimental process as for the PBRB method was executed with six sets of experiments for each method, and each group of experiments was assigned different proportions of training and test sets according to the hly_10879_gr8_bw.tif - method in Section 5.3. The results of the control experiments are calculated by Eq. (25) (26) and (27) and shown in Figures 13, 14, and 15.

## 6 Results and discussion

According to Figure 13, we concluded that the overall accuracy of the PBRB method is highest, followed by the BRB method. The overall accuracy of the other diagnostic methods began to decline rapidly after the 2nd group of tests. The factors contributing to the above results are as follows. First, the PBRB method can better describe the local ignorance in the results compared to the BRB method. Therefore, the overall accuracy of the PBRB method is slightly higher than that of the BRB method. Second, the belief degree in the initial parameters of the PBRB and BRB methods is initialized by expert knowledge, which is closer to the optimal solution of the diagnosis, and the belief degree represents the size of the probability, which is more consistent with the mechanism of WSN. However, the parameters of the other methods are set randomly and aim to fit nonlinear functions as closely as possible, without mechanistic support. Therefore, the model is not as effective as the BRB and PBRB methods for diagnosis. Third, the initial parameters of the PBRB method and BRB method are set in accordance with the mechanism of the diagnosed object. Therefore, it is more suitable for small sample training. However, the other methods fit the approximate nonlinear function by adjusting the parameters, and when the training samples are small, the fitting effect will be affected more, which leads to a sharp decrease in the overall accuracy.

According to Figure 14, PBRB has the highest fault diagnosis accuracy, followed by BRB method. At the same time, fault diagnosis accuracy of the BPNN, KNN, and ELM methods showed a significant decrease in the latter groups of tests. The reasons for the above phenomena are as follows. First, since the PBRB and BRB methods can better handle the ambiguity information in the extracted data features, while the initial parameters set according to the expert knowledge are consistent with the WSN mechanism, they can go for better diagnostic results. Second, because the initial parameters of the BPNN, KNN, and ELM methods are random, their inference process fits a nonlinear function, so the model effect will be significantly decreased when the training samples are small.

According to Figure 15, it can be seen that the PBRB method had the highest fault detection rate, with three 100% detection rates. The detection rates of the other methods were lower than that of the PBRB method. Among them, the detection rate of the KNN method showed a great decrease in the sixth group of experiments. There are several reasons for the above phenomenon. First, the BPNN, KNN, and ELM methods have better fault detection rates in the first five groups of experiments, considering that their overall accuracy and fault detection accuracy show a significant decrease after the third group, which indicates that they have a high error detection rate. Second, the PBRB can effectively handle the information input of fuzzy uncertainty and initial parameter setting in accordance with the mechanism. Therefore, there is no substantial decrease in fault diagnosis accuracy with a high detection rate.

Through the above six groups of comparative experiments, it can be seen that PBRB method has several advantages. Firstly, it can effectively deal with fuzzy uncertain information and the local ignorance caused by it. Secondly, the initial parameters of PBRB model are set according to the expert knowledge, which is more consistent with the WSN mechanism. Finally, the PBRB method can obtain good diagnostic results in the case of small sample training.

## 7 Conclusion

Existing commonly used wireless sensor network fault diagnosis methods have the following problems. First, local ignorance and global ignorance generated by the similarity of fault data features cannot be represented, which affects the diagnosis accuracy of the model. Second, the parameter settings of these methods are random and has no real physical meaning, and the interpretability of the model is poor. Third, the data-driven methods require a large amount of data to train the model to improve the accuracy of the model, and it is difficult to improve the accuracy of the model when the amount of training data is small.

Therefore, a fault diagnosis method of WSN node based on PBRB is proposed. First, local ignorance and global ignorance are represented by a power set. Second, the initial parameters of the model are determined by expert knowledge, which is more consistent with the working mechanism and reduces the dependence on the number of training samples. Third, the P-CMA-ES algorithm is selected to optimize the parameters of the model. In Section 5 of this paper, a case study is constructed to verify the effectiveness of the model. The results of the case study showed that compared with other methods, the accuracy of diagnosis is a small increase. At the same time, better diagnostic results can be obtained with fewer training samples. However, the method proposed in this paper is still in the initial stage and has the following limitations. When the model has more prerequisite attributes or more reference points for the attributes, there is the problem of the explosion of the number of rule combinations. The following research will be carried out in the following aspects.

1. The reasoning process of the PBRB model needs to be optimized to improve the diagnosis accuracy of the model.
2. Some new wireless sensor network node data features need to be extracted, the fuzziness and uncertainty of data features are reduced, and the accuracy of node fault diagnosis is improved.
3. Rule elimination methods need to be proposed to solve the problem of rule combination explosion.

## Declarations

### Author contribution statement

Guo-Wen Sun: Conceived and designed the experiments; Performed the experiments; Analyzed and interpreted the data; Wrote the paper.

Wei He: Conceived and designed the experiments; Analyzed and interpreted the data.

Hai-Long Zhu; Zi-Jiang Yang; Quan-Qi Mu; Yu-He Wangr: Contributed reagents, materials, analysis tools or data.

### Funding statement

Wei He was supported by Postdoctoral Science Foundation of China [2020M683736], 10.13039/501100005046Natural Science Foundation of Heilongjiang Province of China [LH2021F038], innovation practice project of college students in Heilongjiang Province [202010231009; 202110231024; 202110231155], graduate quality training and improvement project of 10.13039/501100013772Harbin Normal University [1504120015], graduate academic innovation project of 10.13039/501100013772Harbin Normal University [HSDSSCX2021-120; HSDSSCX2021-29].

### Data availability statement

Data associated with this study has been deposited at kaggle repository under the accession number Intel Berkeley Research Lab Sensor Data | Kaggle.

### Declaration of interest statement

The authors declare no conflict of interest.

### Additional information

No additional information is available for this paper.
